# Case report: The first case of *Achromobacter xylosoxidans*-related tunnel infection in a patient receiving peritoneal dialysis

**DOI:** 10.1097/MD.0000000000006654

**Published:** 2017-04-21

**Authors:** Jun-Li Tsai, Shang-Feng Tsai

**Affiliations:** a Department of Family Medicine, Cheng Ching General Hospital; b Division of Nephrology, Department of Internal Medicine, Taichung Veterans General Hospital; c Department of Life Science, Tunghai University, Taichung; d Department of Medicine, Nation Yang Ming University, Taipei, Taiwan.

**Keywords:** *Achromobacter xylosoxidans*, peritoneal dialysis, tunnel infection

## Abstract

**Rationale:**

*Achromobacter xylosoxidans* infection is mostly reported in immunocompromised patients. Until now, it is still rarely reported in patients undergoing peritoneal dialysis.

**Patient concerns:**

This is the 1st case of *A xylosoxidans* infection due to tunnel infection of a Tenckhoff catheter.

**Diagnosis:**

The diagnosis was confirmed by the report of culture.

**Interventions:**

Risk factors for this infection in peritoneal dialysis include uremia with an immunocompromised state, contamination due to inexperienced skills, and aqueous environment of the dialysate.

**Outcome:**

We believe that finding the source of A xylosoxidans contamination is the most important aspect of the overall treatment of the infection.

**Lessons:**

Environmental investigation of suspected source contamination is warranted in those with *A xylosoxidans* infection. Once the diagnosis is made, removal of the Tenckhoff catheter should not be delayed.

## 1 Introduction

*Achromobacter xylosoxidans* (*AX*) infection is very rare in patients undergoing peritoneal dialysis (PD). It is mostly detected in immunocompromied patients, such as patients undergoing chemotherapy. Until now, there were only 12 previously reported cases^[1–10]^ of patients receiving PD contracting *AX* infection. Two were an exit site infection^[2]^ and the other 10 cases were peritonitis. Herein, we report the 1st case with *AX* infection due to tunnel infection of a Tenckhoff catheter. We also provide a review of literatures on *AX* infection in PD patients.

## 2 Case report

**Figure 1:**
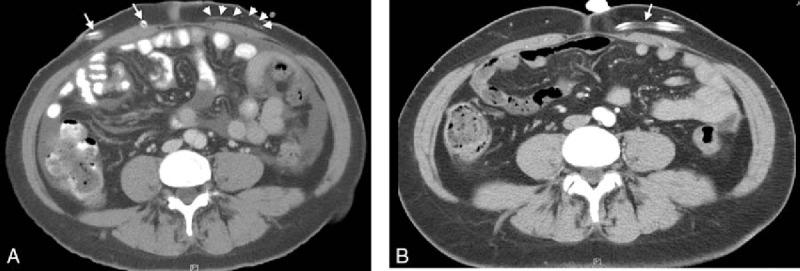
(A) Abdominal tomography for tunnel infection. There is no infection over the new Tenckhoff catheter (white arrow) over her right abdomen, but tunnel infection can be detected over the previous removed Tenckhoff catheter with dirty fat plane (white arrow head). (B) Abdominal tomography 1 year ago. Clean fat plane near the Tenckhoff catheter over left abdomen (white arrow).

A 45-year-old woman had been receiving PD since 5 years ago due to immunoglobulin A nephropathy, and she had no other disease. Her dosage of dialysate was 1.5% of 2L Dianel solution for 4 bags per day. She had never experienced any PD-associated infection in the past 4 years. One year before her 1st hospitalization, she noticed 2 mL of pus around the exit site. After a repeated course of training in PD procedures, symptoms and signs of exit site infection improved. She was afebrile at the time and her dialysate was not cloudy. The pus culture report yielded *Pseudomonas aeruginosa*, which was sensitive to many antibiotics (Gentamicin, Amikacin, Carbapenem, Ceftazidine, Cefepime, Ciprofloxacin, Piperacillin/Tazobactam, and Colistin). Therefore, she received oral Ciprofloxacin for 2 weeks, and the exit site became dry and clean. One month before her 1st hospitalization, she complained of subcutaneous pain along with the Tenckhoff catheter over her left abdomen. There was again no cloudy dialysate and no pain over other abdominal region. She was then hospitalized and received intravenous Amoxycillin/Clavulanate for 2 weeks. However, her condition did not improve. Thus, the surgeon removed the left Tenckhoff catheter and closed the fascia with 2 layers (posterior and anterior sheath). A new Tenckhoff catheter was inserted via her right abdomen. Her dialysate was clean without any turbid materials at the perioperative period. Later, the culture of the removed catheter grew *AX*. This *AX* was sensitive to the following antibiotics, Ampicillin/Sulbactam, Trimethoprim/Sulfamethoxazole, Carbapenem, Cefoperazone 500 mg/Sulbactam 500 mg, Ceftazidine, Piperacillin/Tazobactam, and Tigecycline. This culture was identified by an automated identification system, VITEK 2 system (bioMérieux–Vitek, Hazelwood, MO). She was discharged with oral 3rd-generation Cephalosporin daily and continued to use the new Tenckhoff catheter for PD after discharge. Two weeks after the operation, she was admitted again due to acute onset of abdominal pain along with cloudy dialysate for 3 days. Her white blood cell (WBC) counts of dialysate were 3190/μL. Other examinations of dialysate were as follows: 120 U/L of lactate dehydrogenase, 0.35 g/dL of total protein, and 450 mg/dL of glucose. Her blood tests were 12,000/cumm of WBC, 89 mg/dL of blood urea nitrogen, 12.2 mg/dL of serum creatinine, 3.5 mg/L of C-reative protein, 110 mg/dL of blood sugar, 120 mg/dL of low-density lipoprotein cholesterol, and 200 mg/dL of triglyceride. Her blood and urine cultures were both negative. Therefore, PD-related peritonitis was diagnosed. Empirical antibiotics with 1st- and 3rd-generation Cephalosporins (Cefazolin and Ceftazidime, 125 mg/L) were infused via irrigation. Three days after the infusion of antibiotics, the WBC of dialysate dropped to 1729 and 169/μL, and the dialysate became clean and colorless. Finally, the culture of dialysate yielded *AX*. The antimicrobial susceptibility testing of *AX* during this 2nd admission was the same as 2 weeks ago from the removed catheter suggesting the same pathogen as the cause. The test showed that the pathogen was resistant to 1st-generation Cephalosporin and Ciprofloxacin, but sensitive to Ceftazidime and Ampicillin/Sulbactam. The abdominal tomography disclosed dirty fat planed over the previous tunnel (Fig. 1B), compared with the previous a year ago (Fig. 1A). Finally, the final diagnosis was inadequate treatment for *AX*-related tunnel infection, which progressed to PD-related peritonitis. Since the dialysate became clean and there was no more abdominal pain, she was discharged with oral Ampicillin/Sulbactam for 2 more weeks. The total 3-week antibiotics courses were applied and she recovered well. This study had been approved by the patient herself and she signed the informed consent.

## 3 Discussion

*Achromobacter xylosoxidans* (formerly *Alcaligenes xylosoxidans*) is a nonfermenting, aerobic, oxidase- and catalase-positive, and gram-negative rod with peritrichous flagella. Normally, it is distributed in aqueous environments including soil, water, rotten eggs, and dairy products. In human bodies, it may also exist as normal flora over skins and in the gastrointestinal tract. However, in immunocompromised patients such as patients undergoing chemotherapy, it could cause pneumonia, pharyngitis, catheter-related infection, external otitis, and urinary tract infections. Until now, there were only 12 reported cases of *AX*-related infection in PD^[1–11]^ (Table 1). Of them, most (10 cases) were PD-related peritonitis, and 2 were an exit site infection.^[2,11]^ Our case is the 13th case of *AX*-related infection in PD but it is the 1st case with *AX*-related PD tunnel infection. Of all 13 cases with *AX*-related PD infection, the mean age was 46.2-year old, and 61.5% were female. Almost half (41.6%) of the PD cases were due to glomerular disease, followed by diabetic nephropathy (33.3%). Most cases (53.8%) recovered after the removal of catheters. This was because *AX* may form biofilm on plastic materials like PD catheters and was difficult to be eradicated without the removal of catheters.^[3]^ Keeping PD catheters and manually flushing them could mechanically shear the biofilm and cause detachment of cells or aggregates. Therefore, we suggested strict removal of PD catheters immediately if a diagnosis of *AX*-related infection was made.

**Table 1:**
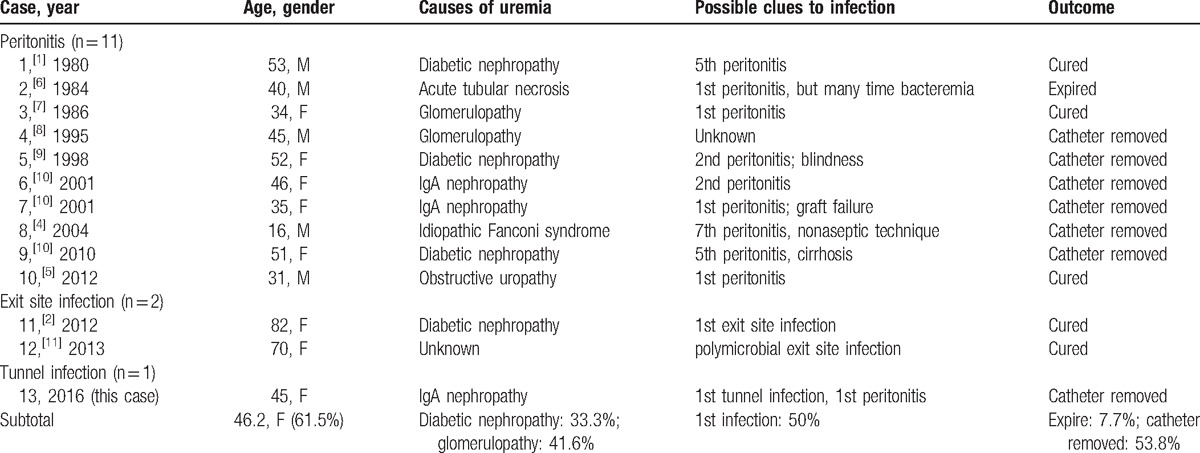
Reported cases with of *Achromobacter xylosoxidans*-related peritoneal dialysis infection.

The virulence of *AX* is considered to be weak, so it commonly infects immunocompromised patients. There are some characteristics for PD favoring the growth of *AX*. Firstly, renal failure confers the immunocompromised state. Secondly, glucose containing dialysate provides an aqueous environment with much nutrition for *AX* growth. As in case 9, cirrhosis also made for a more immunocompromised state.^[3]^ However, we believed that there are still some reasons predisposing *AX* infection in PD because reports of *AX* infection in PD were still rare. One reason was the inexperienced skills for PD (such as case 5 with blindness^[9]^). Half of the *AX* infections in PD were more than 1 peritonitis (5 times peritonitis for 2 cases and 7 times peritonitis for 1 case^[4]^). Finally, *AX* contaminated water without thorough disinfection was also the major problem. We should bear in mind the possibility of contaminated sources if the occurrence of *AX*-related peritonitis was in more than 1 patient. Unfortunately, there was no clear source of *AX* infection in all 13 cases, except for our patient. We performed environmental investigation cultures from available open solutions in her house. Then a positive culture for *AX* was detected in a tap water sample from her faucet. According to the antibiotic susceptibility, *AX* from her PD catheter and her dialysate may came from the tap water from the faucet. Our patient claimed that she did not regularly wash her hands thoroughly with disinfectants. She also admitted to touching her PD catheter after washing hands with tap water without using disinfectants. That can explain why she had *AX*-related tunnel infection, followed by *AX*-related peritonitis. This is the 1st case of *AX*-related PD infection with clear environmental investigations. Our investigation helps to understand the mechanisms of *AX*-related PD infection. It provides a better understanding of *AX* in the environments causing infection in PD.

## 4 Conclusion

*AX*-related infection is still rare currently but we still should be reminded to remove PD catheters if this diagnosis is confirmed. Meticulous environmental investigation can guide clinicians to avoid recurrent *AX* infection. Despite its rarity, we should not delay the diagnosis of this disease, especially in high-risk patients.

## References

[1] Igra-Siegman Y Chmel H Cobbs C Clinical and laboratory characteristics of *Achromobacter xylosoxidans* infection. J Clin Microbiol 1980;11:141–5. 7358838 10.1128/jcm.11.2.141-145.1980 PMC273340

[2] Tsai MT Yang WC Lin CC Continuous ambulatory peritoneal dialysis-related exit-site infections caused by *Achromobacter denitrificans* and *A. xylosoxidans*. Perit Dial Int 2012;32:362–3. 22641747 10.3747/pdi.2011.00207 PMC3525435

[3] Tsai SF Shu KH CAPD peritonitis caused by *Alcaligenes xylosoxidans* in a diabetic cirrhosis patient. Ren Fail 2010;32:899–901. 20662708 10.3109/0886022X.2010.494796

[4] Melgosa M Espinazo O Alonso A Dialysis-associated *Alcaligenes xylosoxidans* peritonitis: a pediatric case. Perit Dial Int 2004;24:72–5. 15104341

[5] Kocak G Azak A Huddam B Continuous ambulatory peritoneal dialysis-related peritonitis in an uremic outpatient: *Achromobacter xylosoxidans*. Blood Purif 2012;34:1–2. 22814104 10.1159/000337879

[6] Reverdy ME Freney J Fleurette J Nosocomial colonization and infection by *Achromobacter xylosoxidans*. J Clin Microbiol 1984;19:140–3. 6699141 10.1128/jcm.19.2.140-143.1984 PMC271003

[7] Morrison AJ Jr Boyce Kt Peritonitis caused by *Alcaligenes denitrificans* subsp. *xylosoxydans*: case report and review of the literature. J Clin Microbiol 1986;24:879–81. 3771775 10.1128/jcm.24.5.879-881.1986 PMC269052

[8] Haqqie SS Roth M Bailie GR Unsuccessful treatment of CAPD peritonitis caused by *Alcaligenes xylosoxidans* subsp. *denitrificans*. Ren Fail 1995;17:611–4. 8570874 10.3109/08860229509037626

[9] El-Shahawy MA Kim D Gadallah MF Peritoneal dialysis-associated peritonitis caused by *Alcaligenes xylosoxidans*. Am J Nephrol 1998;18:452–5. 9730575 10.1159/000013370

[10] Tang S Cheng CC Tse KC CAPD-associated peritonitis caused by *Alcaligenes xylosoxidans* sp. *xylosoxidans*. Am J Nephrol 2001;21:502–6. 11799270 10.1159/000046657

[11] Lew SQ Gruia A Ofloxacin solution for persistent exit-site and tunnel infection in peritoneal dialysis. Perit Dial Int 2013;33:101–2. 23349200 10.3747/pdi.2012.00070 PMC3598270

